# The Northwestern Monthly for July

**Published:** 1897-06

**Authors:** 


THE NORTHWESTERN MONTHLY for July will be a special number, and will contain a series of excellent articles upon the care of the child. It will contain five special sections upon the "physical child"—exercise, defects, care of the body, the school building, general lessons, systems, etc. These different points will be brought out by prominent writers like Superintendent G. E. Johnson, of Andover, Mass.; Miss G. Sisson, of Leland Stanford University; Dr. R. A. Clark, Dr. Oscar Chrisman, Dr. H. K. Wolfe, and others.

"Medical Inspection of Schools" is an article contributed by Dr. C. F. Menninger, the leading homeopathic physician of Topeka, Kansas, and President of the Kansas State Society, and member of Kansas State Board of

Health, who wrote the article entitled "The Conquest," published in THE HOMEOPATHIC PHYSICIAN for July, 1896, page 304. Price of this special number, 25 cents. Address J. H. Miller, Publisher, Lincoln, Nebraska.

